# Blockchain technology and raft consensus for secure physician prescriptions and improved diagnoses in electronic healthcare systems

**DOI:** 10.1038/s41598-024-66746-y

**Published:** 2024-07-08

**Authors:** Behnaz Abdorrahimi, Atefeh Nekouie, Amir Masoud Rahmani, Jan Lansky, Vladimír Nulíček, Mehdi Hosseinzadeh, Mohammad Hossein Moattar

**Affiliations:** 1 Electrical Engineering Department, Imam Reza International University, Mashhad, Iran; 2 Department of Computer Engineering, Mashhad Branch, Islamic Azad University, Mashhad, Iran; 3 Future Technology Research Center, National Yunlin University of Science and Technology, Yunlin, Taiwan; 4 Department of Computer Science and Mathematics, Faculty of Economic Studies, University of Finance and Administration, Prague, Czech Republic; 5 Institute of Research and Development, Duy Tan University, Da Nang, Vietnam; 6 School of Medicine and Pharmacy, Duy Tan University, Da Nang, Vietnam

**Keywords:** Electronic healthcare system, Blockchain, Consensus algorithm, Transparency, Security, Engineering, Health services

## Abstract

With electronic healthcare systems undergoing rapid change, optimizing the crucial process of recording physician prescriptions is a task with major implications for patient care. The power of blockchain technology and the precision of the Raft consensus algorithm are combined in this article to create a revolutionary solution for this problem. In addition to addressing these issues, the proposed framework, by focusing on the challenges associated with physician prescriptions, is a breakthrough in a new era of security and dependability for the healthcare sector. The Raft algorithm is a cornerstone that improves the diagnostic decision-making process, increases confidence in patients, and sets a new standard for robust healthcare systems. In the proposed consensus algorithm, a weighted sum of two influencing factors including the physician acceptability and inter-physicians’ reliability is used for selecting the participating physicians. An investigation is conducted to see how well the Raft algorithm performs in overcoming prescription-related roadblocks that support a compelling argument for improved patient care. Apart from its technological benefits, the proposed approach seeks to revolutionize the healthcare system by fostering trust between patients and providers. Raft’s ability to communicate presents the proposed solution as an effective way to deal with healthcare issues and ensure security.

## Introduction

Healthcare data is a vital component of the medical field, encompassing a wide spectrum of information related to patients, medical conditions, treatments, research, and more. It plays a fundamental role in providing effective healthcare services, making informed medical decisions, conducting research, and improving patient outcomes^1^. This information may face various security threats such as patient privacy breaches, data integrity, and identity verification issues. This is because these data are stored in centralized databases, and the loss of this data could disrupt the process of diagnosis and improvement of diseases. Optimizing diagnostic and treatment methods is a priority for healthcare systems in all societies^2^. Due to the high importance of preserving patient privacy in electronic health records, there is a need for secure methods for data storage and processing, as these data contain sensitive personal and medical information^3^. Striking a balance between providing authorized healthcare professionals with access and maintaining patient confidentiality is crucial^4^.

In this age of unprecedented technology advancements, a dramatic overhaul of the healthcare sector is imminent. Due to its data immutability feature, blockchain technology can be applied in an integrated network for distributed data capturing, storing, and sharing^5^. A noteworthy use of blockchain technology is the secure data interchange between medical professionals. This technology supports pharmaceutical versions, supply chain management, access control, and data exchange in the healthcare area, among other difficulties that the industry faces. Healthcare solutions built on the blockchain can improve patient data security and dependability. The sharing of prescription data between many parties, including doctors, chemists, and insurance companies, is made easier by blockchain interoperability. According to^4^, this straightforward communication lowers the administrative load and boosts the healthcare system’s general efficiency.

However, to fully harness the potential of blockchain in healthcare, it’s essential to integrate robust consensus mechanisms that ensure data accuracy and security. With the introduction of blockchain technology, consensus mechanisms can be digitally facilitated and automated through the use of a distributed ledger^6^. The decentralized nature of blockchain allows transparent and secure sharing of version information between authorized participants. Network users cooperate to verify and log each version entry using consensus techniques like proof-of-work (PoW) or proof-of-stake (PoS). Version information is trusted thanks to this digital consensus, which also prevents changes without permission from authorized parties.

Doctors are driven to engage in diagnostic consensus because their dedication to patient well-being compels them to strive for precise diagnostics, thereby improving patient outcomes. Secondly, they maintain professional standards by promoting evidence-based approaches. Moreover, doctors are morally obligated to endorse dependable diagnostics, in line with their duty to patients and the medical field. The process of collaborative validation among healthcare stakeholders to assure the validity and reliability of recommended therapies, improve patient safety, and increase healthcare efficiency is generally embodied by consensus in medical prescriptions^7^.

In the continually evolving landscape of healthcare, a significant challenge revolves around optimizing the recording and management of physicians’ prescriptions a fundamental element of medical services. This article presents an innovative solution by leveraging blockchain technology and the Raft consensus algorithm, highlighting their transformative potential. The Raft algorithm specifically tackles challenges associated with physicians’ prescriptions, elevating the security and reliability of recommendations, and thus enhancing the medical decision-making process. Via a thorough analysis, it is shown how Raft excels in addressing prescription-related challenges, offering a prescription for refined diagnoses and elevated patient care.

RAFT, a consensus algorithm designed for distributed systems, focuses on ensuring consensus among a cluster of nodes. It achieves this by electing a leader among the nodes, which then coordinates the replication of logs across the cluster. RAFT is known for its simplicity and understandability, making it a popular choice for systems where ease of implementation and maintenance is crucial. In the context of healthcare, RAFT enhances the security and reliability of recommendations, crucial for accurate medical decision-making. By ensuring that all nodes agree on the state of the system, RAFT minimizes the risk of inconsistencies or errors in prescription management, ultimately leading to improved patient care.

Blockchain technology, on the other hand, provides a decentralized and immutable ledger for recording transactions or data. It ensures transparency, security, and tamper-resistance by storing data across a distributed network of nodes in a chronological chain of blocks. In healthcare, blockchain can be used to securely store patient records, prescriptions, and other sensitive information, reducing the risk of data breaches or unauthorized access.

Comparing RAFT with other consensus algorithms, such as Paxos or Practical Byzantine Fault Tolerance (PBFT), RAFT stands out for its simplicity and ease of understanding. While Paxos and PBFT are highly efficient in certain scenarios, they often come with a steeper learning curve and more complex implementation requirements. RAFT’s straightforward approach makes it more accessible, especially for systems where robustness and reliability are paramount, like healthcare applications.

This strategic approach aims to revolutionize the healthcare industry, fostering trust among both patients and healthcare providers alike. Furthermore, in confronting the intricate and sensitive issues inherent in healthcare, the article underscores the heightened importance of safeguarding patient information. By capitalizing on blockchain technology and the Raft consensus algorithm, the article puts forth solutions to fortify the security and scalability of patient data. This technological integration contributes significantly to advancing data security in the healthcare sector, cultivating increased trust among patients and service providers. The amalgamation of blockchain and Raft consensus emerges as a transformative prescription poised to revolutionize healthcare and enhance the lives of countless individuals.

Also, maintaining the mindset of the main doctor’s diagnosis proposal in prescription sharing through the RAFT algorithm includes designating the main doctor as the leader node, empowering them to initiate proposals and guide the agreement process. Other peers are invited by the lead physician to review and vote on the proposal, but the leader’s vote carries more weight. The RAFT algorithm ensures that consensus is reached between nodes and that the input of the primary physician influences the final decision. Transparent record-keeping on the blockchain facilitates accountability and traceability and ensures that the mindset of the primary physician diagnosis remains integrated within the common framework of prescription sharing.

## Related work

### Consensus protocol

Within the blockchain network, there is no central hub or governing authority that dictates the sequence of transactions, validates them or establishes communication protocols among nodes. Transactions and documents are transparent and traceable. In addition to the different forms and approval protocols that have been introduced, consensus ensures that a quorum of nodes reaches an agreement on the precise order of new entries on the shared ledger^8^.

Consensus is a crucial process for validating the timing of request completion, modification, or development, as well as transactions (deployment and invocation) and their associated details. Correct sequencing is of paramount importance because it establishes ownership, additional rights, and obligations. In simpler terms, consensus is the mechanism that ensures everyone on the network agrees on the order and validity of transactions, providing transparency and reliability in a decentralized system^9^. In this section, a classification of different consensus protocols used in blockchain^10^ is presented.

Proof of Work (PoW): Bitcoin is an example of a consensus protocol used in blockchain networks. A new block can be added to the blockchain by miners, who compete to solve challenging mathematical puzzles. The first person to solve a puzzle gets to add it to the blockchain. Because it is impractical for a single entity to dominate the network due to the computational power necessary to answer these problems, PoW ensures decentralization and network security. PoW does, however, have certain disadvantages, such as high energy consumption and scaling issues^10^.

Proof of Stake (PoS): This technique chooses block validators based on the quantity of cryptocurrency they are prepared to "stake" as collateral, in contrast to Proof of Work (PoW). Validators are selected in a pseudorandom or predictable manner to generate new blocks. Energy efficiency is provided by PoS, which is frequently seen to be more scalable than PoW. This protocol does have many drawbacks, though, including worries about possible centralization and the "Nothing at Stake" issue, which allows validators to accept numerous forks without suffering repercussions^11^.

Practical Byzantine Fault Tolerance (PBFT) is a consensus mechanism designed for distributed systems that are susceptible to Byzantine faults, which are defined as instances in which a certain percentage of nodes may be hostile or dysfunctional. Through a multi-round procedure in which nodes exchange messages, consensus is reached. When a value is accepted by at least two-thirds of the nodes, consensus is reached. Despite its reputation for speed and finality, PBFT requires node identification and may require a fixed set of nodes, which could lead to centralization^12^.

Raft: Raft is a consensus algorithm aimed at ensuring fault tolerance in distributed systems. It elects a leader among nodes responsible for managing the replication of the log across all nodes. If a leader fails, a new leader is elected. Raft is praised for its simplicity, making it more accessible to implement and understand than some other consensus protocols like PBFT^3^. While it can tolerate nodes crashing and recovering, Raft has limitations on fault tolerance, assuming that no more than half of the nodes can fail simultaneously, and it may be slower than some alternatives like PBFT in certain scenarios^13^.

### Blockchain technology in healthcare data

With the widespread application of information technology in the medical field, the volume of generated medical data has increased. Given the substantial amount of data with diverse structures, collecting and analyzing this data, as well as exploring their potential value, can effectively advance clinical medicine and pharmaceutical research and development^14^. Therefore, designing a data-sharing and medical record-sharing solution among physicians and various stakeholders that can safeguard patient privacy while allowing data analysis is of paramount importance. Numerous solutions have been proposed for protecting the privacy of medical data. In the data acquisition stage, traditional privacy-preserving approaches are primarily based on anonymization techniques. In modern healthcare, information sharing and collaboration among specialized physicians play a crucial role in providing comprehensive patient care. However, ensuring the security, privacy, and accuracy of patient data while sharing them among multiple specialists can be a challenging task. This is where blockchain technology, coupled with a consensus algorithm, can offer a robust solution^15^. Blockchain technology has transformed the healthcare ecosystem with its distinct features including decentralization, security, immutability, stability, anonymity, and auditability^16^. Blockchain has the potential to reshape the traditional sharing of Electronic Health Records (EHRs) across multiple healthcare institutions, enhancing the quality, intelligence, and efficiency of healthcare. By leveraging blockchain and consensus algorithms in this approach, healthcare professionals can collaborate seamlessly while maintaining the security, accuracy, and transparency of data. Patients benefit from a higher level of care coordination among specialists, leading to improved treatment outcomes and overall healthcare experiences. In^17^, blockchain has been utilized for management and sharing within healthcare systems. In its initial version, MedRec addressed heterogeneous data, collaboration capabilities, patient-centricity, and research data in healthcare. It also employed the Proof of Work consensus protocol for securing the healthcare system. However, the computational cost of PoW increased as network participants grew. In^18^, blockchain technology has been used to securely store and share sensitive patient data, streamline administrative processes, and enhance healthcare outcomes. It also employs a multi-leader consensus protocol for its operation. This protocol allows multiple nodes to validate and add transactions to the blockchain concurrently, improving the scalability and fault tolerance of a blockchain network and making it more suitable for large-scale healthcare systems. In^19^, a blockchain-based system is proposed for secure and efficient management of healthcare data. The proposed system utilizes the Practical Byzantine Fault Tolerance (PBFT) consensus algorithm to ensure the integrity and consistency of stored data in the blockchain. Additionally, this system employs lightweight blockchain technology to reduce storage and computational costs associated with traditional blockchain systems. In^20^, a multi-tiered blockchain-based sharing scheme is employed, and their workflow is based on the Proof of Work consensus mechanism.

In^21^ proposed the inclusion of a reputation model within the conventional PBFT consensus mechanism for the identification of malicious nodes and the selection of the primary node. Nodes with superior reputations stand a greater chance of being chosen as the primary node. The suggested model underwent testing in a simulated environment, with experimental results showing enhanced power, reduced latency, and a decreased rate of faulty nodes in comparison to traditional PBFT, signifying an enhancement in system performance. Nonetheless, the incorporation of supplementary data structures in the proposed model leads to performance overhead, necessitating the consideration of optimization techniques for future implementation in a blockchain environment.

In^22^, the authors explored the use of distributed reinforcement learning in a Federated Learning system and the advantages of integrating Blockchain Technology. Their method aims to enhance clinical monitoring and ensure secure communication and data privacy in a decentralized manner. Some other works done in this field are briefly mentioned in Table 1. Besides, a comprehensive review of cloud healthcare services and be found in^23^.

**Table 1:** Summary of related work.

References	Solutions	Consensus Algorithm	Disadvantage
^19^	providing services ensuring security and maintaining the privacy of patients	Practical Byzantine Fault Tolerance (PBFT) consensus algorithm	Increased bias, manipulation risks, low reliability
^24^	Traceability of consent in clinical trials	Proof of concept with time stamp	Unsure whether the person signed the consent is the right one or not
^25^	Tracking, securing, and management of clinical trials	Permissioned Ethereum, blockchain	Lack of network scalability
^26^	Monitoring and management of clinical data in multisite trials	Permissioned Hyperledger Fabric, blockchain	The cost of network setup is high
^27^	Monitoring and detecting falsified, spurious and counterfeit drugs	Ethereum Hyperledger Fabric, and Delegated PoS (DPoS) and practical Byzantine fault tolerance (PBFT	It requires implementation plans and policies
^28^	Inspection and tracking the data flow of drugs to prevent counterfeit drugs	Consortium PoW	Consultation with key investors to perform a cost–benefit evaluation
^29^	Analyze, trace, manage and verify medical data for clinical trial and precision medicine	General blockchain platform	Lack of consistency
^21^	Identification of malicious nodes and the selection of the primary node	PBFT consensus mechanism	Nodes with a lower value cannot participate in the consensus at all and it causes bias
^30^	Improve the scalability and throughput	MBFT consensus mechanism	Using the two-layer consensus algorithm increases complexity, requires more resources
^31^	Reducing the overhead for validating and recording transactions	Geographic-PBFT	Inequality of resources, increasing complexity
^22^	Improve clinical monitoring, ensure secure communication, and enhance data privacy in a decentralized manner	–	Complexity, Scalability, Security Risks

## System model

In this section, the proposed consensus approach among doctors using the RAFT algorithm is explained, which provides more security and reliability between patients and doctors.

The Raft consensus algorithm is favored for medical documents and doctors’ consensus due to its simplicity, reliability, and fault tolerance. Unlike more complex algorithms, Raft is designed for easier implementation and comprehension, making it accessible to healthcare professionals who may not have specialized technical knowledge. Its strong consistency guarantees that all nodes agree on the state of data, crucial for accurate medical records and doctors’ decisions. Raft’s fault tolerance capabilities ensure system reliability, crucial in healthcare where uninterrupted access to data is vital. Additionally, Raft’s adaptability to permissioned networks aligns well with the controlled environment of healthcare organizations, allowing for efficient consensus among trusted parties without the complexities of permissionless networks. Overall, Raft’s simplicity, reliability, and adaptability make it a preferred choice for ensuring consensus in medical settings. Table 2 compares different consensus methods.

**Table 2:** Comparison on consensus algorithms.

Feature	Raft	PoW	PoS	PBFT	DPoS
Consensus mechanism	Leader-based	Computational power (mining)	Staking (based on ownership)	Byzantine fault tolerance	Delegated staking (voting)
Leader election	Leader election based on timeout mechanism	N/A	N/A	Distributed agreement	Delegated (voted by stakeholders)
Economic Incentives	None	Block rewards (mining)	Staking rewards	None	Block rewards and voting rewards
Fault tolerance	Limited faults tolerated	Tolerates some network faults	Tolerates some network faults	Tolerates Byzantine faults	Tolerates Byzantine faults
Scalability	Suitable for smaller networks	Limited scalability	Moderate scalability	Moderate scalability	Moderate scalability
Complexity	Simple and easy to understand	Complex and resource-intensive	Moderate complexity	Moderate complexity	Moderate complexity
Use cases	Suitable for systems requiring strong consistency and reliability, such as electronic healthcare systems	Public blockchains, where decentralization and security are paramount	Public and private blockchains, where energy efficiency and scalability are important	Distributed systems requiring Byzantine fault tolerance, such as financial systems	Blockchains where efficient governance and scalability are desired

### System architecture

**Figure 1:**
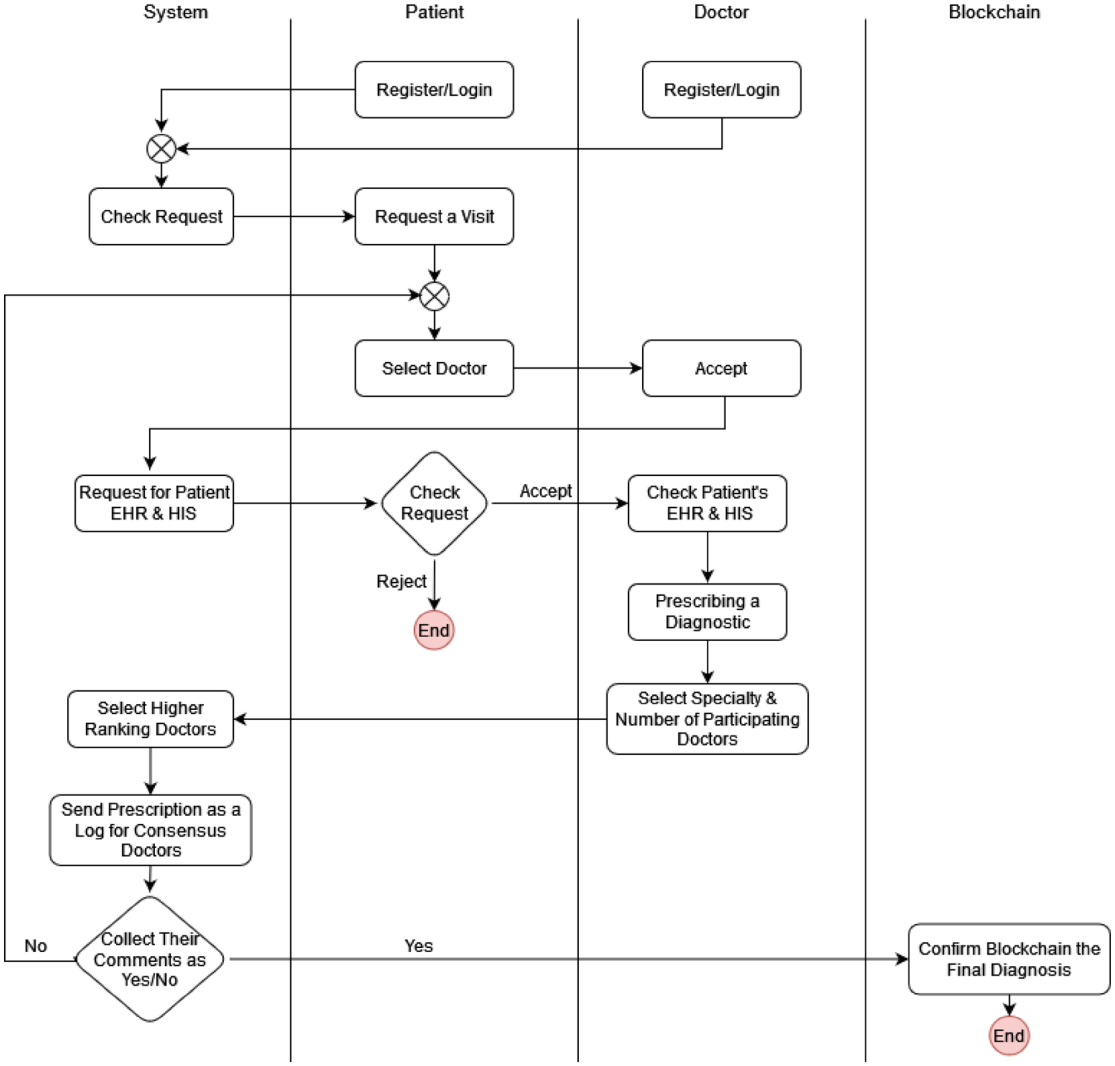
Proposed Model.

The initial stage of the proposed method’s architecture is visually depicted in Fig. 1. This pioneering approach introduces a state-of-the-art method for delivering medical and healthcare services while promoting consensus among specialized physicians. Each participating physician has the opportunity to contribute their expert assessment of a patient’s diagnosis, considering the patient’s comprehensive medical and treatment history, as well as the diagnosis provided by the treating physician. The patient is ultimately prescribed the diagnosis with the highest consensus among the medical experts, and all versions of prescriptions are securely stored within immutable blocks, ensuring the prevention of any unauthorized alterations.

### Consensus steps

*Step 1* Choosing the Treating Physician

Phase 1-1: Registration of Physicians in the System.

Each physician, during the registration process in the system, enters their personal and professional information (medical credentials). The system verifies the accuracy of the physician’s information through validation. The result of the validation process is communicated to the individual in the form of a message.

Phase 2-1: Patient Registration in the System.

Every patient is required to complete their registration in the system by providing their personal information. The system then verifies the accuracy and authenticity of the provided data through a validation process.

It is noteworthy that patients, when engaging with any physician and undergoing their treatment process, have the opportunity to share their feedback on the diagnosis, the physician’s approach, and other aspects of the interaction, regardless of the final outcome. All these valuable opinions are recorded in the system, allowing it to calculate an average rating and rank physicians accordingly. The average user ratings provided by patients in the system serve as a significant parameter for the statistical ranking of physicians.

Phase 3-1: Choosing the Attending Physician by the Patient.

After logging into the system, the patient indicates the medical specialty they would like to pursue. Next, the system shows the patient a list of licensed doctors from which they can choose their favorite medical professional. The patient selects a candidate from the list to serve as their treating physician. The rankings that other patients have given in the physician evaluation are also displayed to help the patient make an informed choice. In the suggested algorithm, the chosen doctor takes the lead position. The patient informs the chosen healthcare practitioner about their ailment or condition after choosing the attending physician.

*Step 2* Formation of Blocks.

Phase 1-2: Submission of the patient’s medical record to the treating physician by the patient.

Once the patient chooses the treating physician, they send a request to access their medical records, and upon the patient’s confirmation, they forward their medical history to the selected physician.

Phase 2-2: Choosing Physicians to Participate in Consensus.

**Figure 2:**
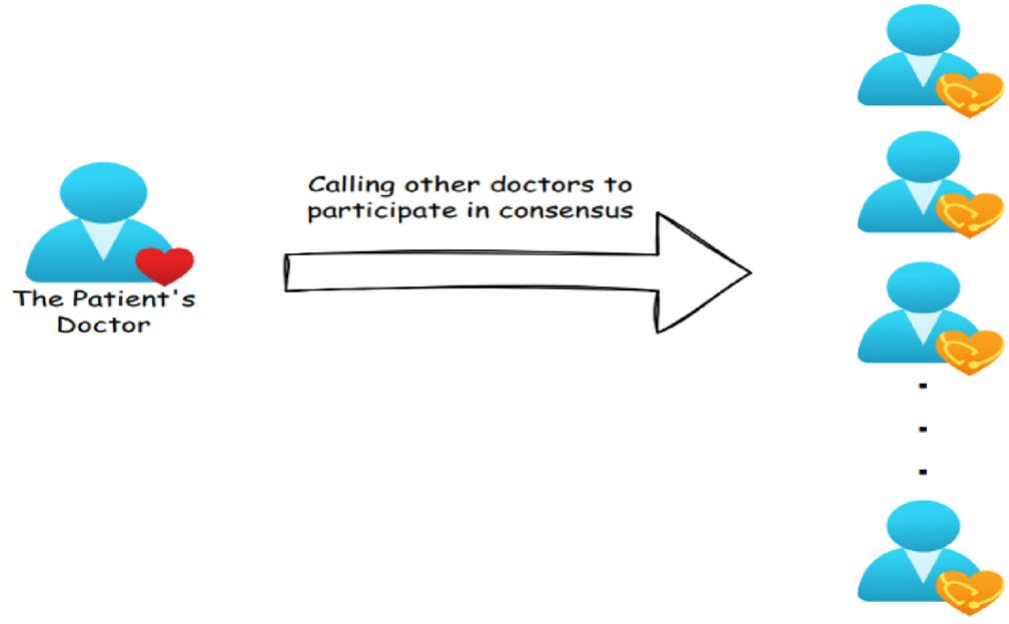
The treating physician initiates the request for selecting doctors to participate in the consensus.

Once the treating physician reviews the patient’s medical records and their statements, they submit a request through the system to select other physicians for participation in the consensus process, as shown in Fig. 2. Subsequently, the system presents a list of eligible physicians to the treating physician, who can then send them invitations to join the consensus based on their respective specialties. The treating physician has the flexibility to invite a specific number of physicians to participate in the consensus.

The selection of additional physicians will be carried out by the system using the following formula.

Weighted Sum Formula for specialist selection:

1 $$C_{i} = \alpha S_{1i} + \left( {1 - \alpha } \right)S_{2i}$$

The selection of participating physicians in the consensus is based on the weighted sum of two criteria. This parameter is denoted as C_i_ for the ith physician in this article and is computed as Eq. (1). One of the criteria (S_1i_ for physician i) is the average ratings of users, i.e., patients, regarding their interactions with the physician i and the effectiveness of the treatment process.

The second criterion denoted as S_2i_, is the degree of agreement of the ith physician’s decisions with other physicians. This criterion is measured using the kappa index, which is computed as Eq. (2). The higher the level of agreement, the higher the physician’s ranking.

2 $$S_{2i} = \frac{{\mathop \sum \nolimits_{{\begin{array}{*{20}c} {j = 1} \\ {j \ne i} \\ \end{array} }}^{N} \left( {P_{ij} \left( A \right) - P_{ij} \left( E \right)} \right)/ (1 - P_{ij} \left( E \right)}}{N - 1}$$

In which ${P}_{ij}\left(A\right)$ is the actual proportion of agreements between physicians i and j, ${P}_{ij}\left(E\right)$ is the probability of causal agreement and N is the total number of physicians. Also, in Eq. (1),$\alpha$ is the weighting parameter and can be according to the coordinator preferences.

Phase 3-2: The treating physician (leader) submits their comments and the medical records.

**Figure 3:**
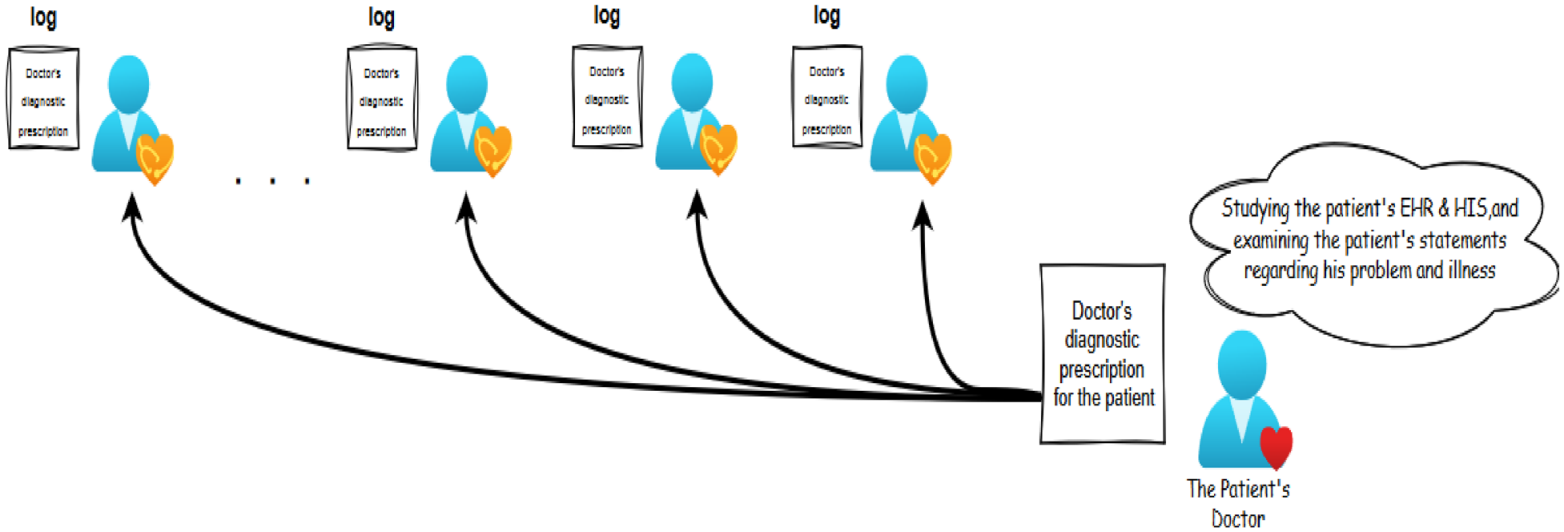
Sending a copy of the diagnostic version to the doctors participating in the consensus.

The treating physician, who also assumes the role of the leader, examines the patient’s medical records, provides their assessment, and records it in a block. After that, as shown in Fig. 3, Subsequently, the block is sent to other physicians for verification, with each of them having a copy of the block. The selection of the treating physician by the patient and the consensus among other physicians are facilitated using the proposed method based on the Raft algorithm.

Phase 4-2: Voting.

Other physicians, after reviewing the patient’s medical records and the medical issue, express their opinions and cast their votes (yes/no) in the log. If the treating physician’s opinion receives a majority of votes, it will be approved. However, if it gets rejected, the entire consensus process must be repeated with a new leader.

Benefits of Using Blockchain: Since the system is connected to the blockchain network, all of these activities will be securely recorded in the blockchain. This ensures that no physician or individual can manipulate or delete their opinions or diagnoses. The significance of this lies in the fact that any incorrect or inaccurate diagnosis by a physician could lead to further issues for the patient, causing complications and potentially allowing the physician to avoid accountability by altering previous instructions or prescriptions in their favor.

To prevent such tampering and enhance the accountability of physicians for their diagnoses, all opinions and diagnoses must be faithfully recorded in blockchain blocks. This way, in case of any problems arising from incorrect diagnoses, the patient can easily trace and investigate the matter. Furthermore, the consensus among physicians ensures that if any medical aspect has been overlooked or not adequately addressed in the patient’s medical records, it can be thoroughly reviewed during the consensus process. This empowers the patient to pursue a better and more reliable path towards recovery.

## Evaluation and discussion

**Figure 4:**
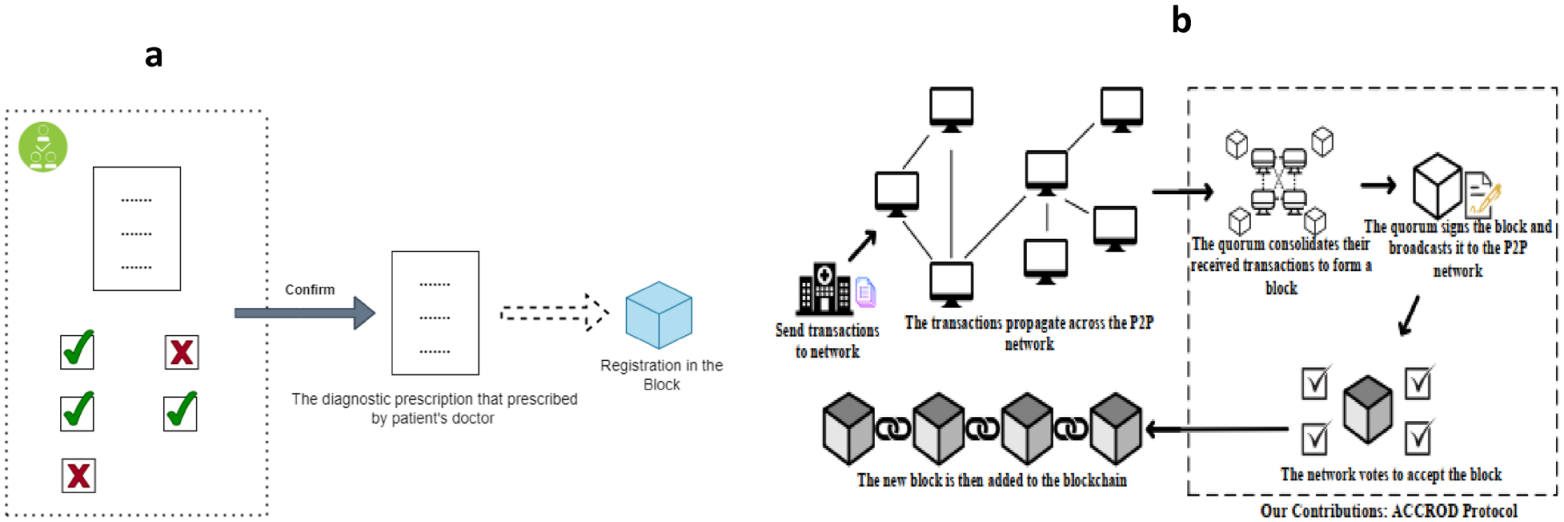
(**a**) Comparing the architecture of the proposed method with (**b**) the architecture of the previous work^18^.

In^18^, the discussed architecture is that of a multi-leader system, where leaders are randomly selected, as depicted in Fig. 4b, which is not suitable for healthcare and medical research. Given the nature of healthcare studies, the patient must have a transparent role in their treatment process. In the proposed method (Fig. 4a), the patient selects their leader or treating physician, and the opinions of other participants in the consensus (other doctors) are evaluated based on the leader’s judgment. Furthermore, in the proposed method, the selection of other participants to join the consensus process is not random but is determined using a Weighted Sum formula.

**Figure 5:**
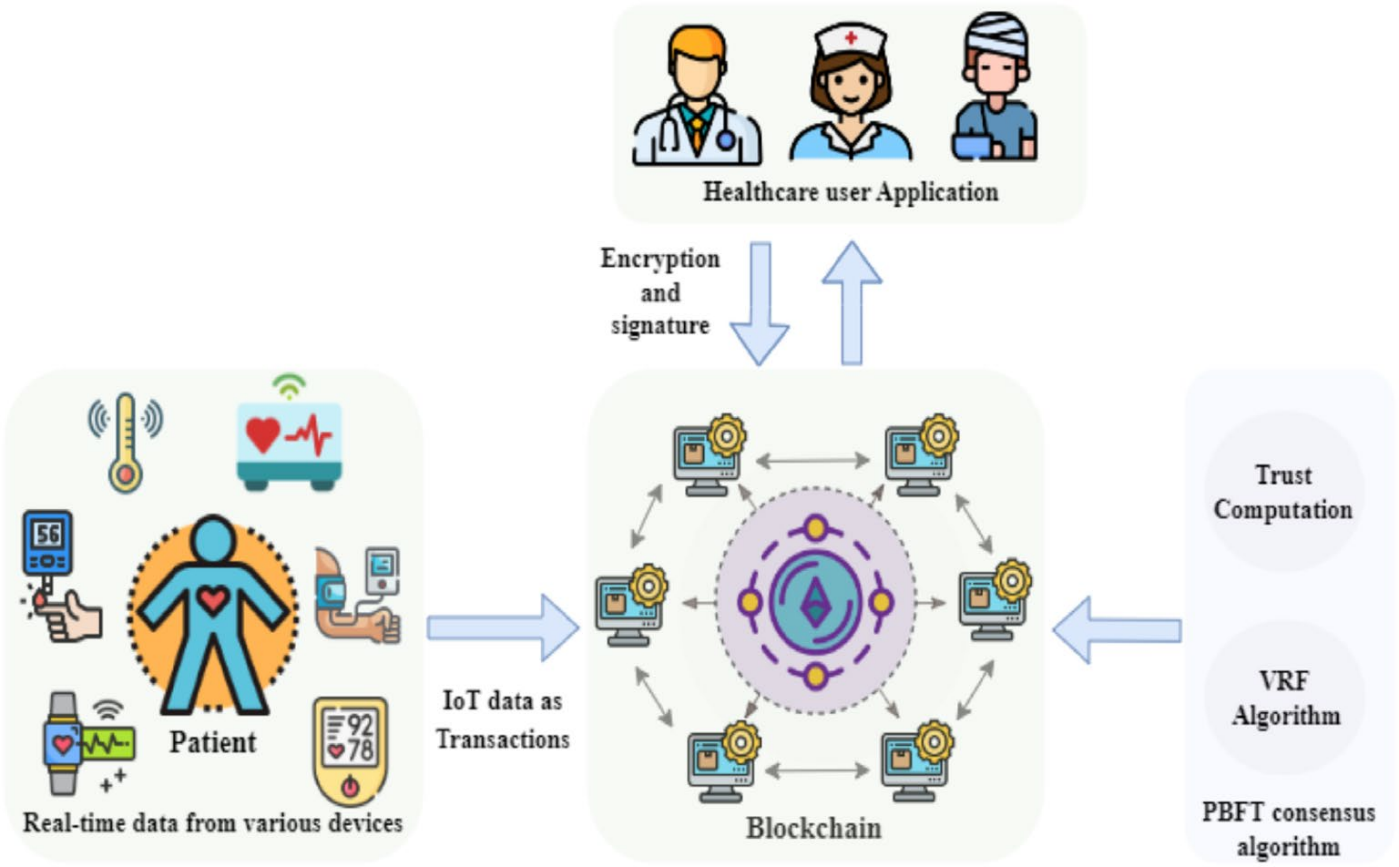
Architecture used in healthcare^19^.

This paper proposes a lightweight consensus-based PBFT blockchain for healthcare applications by incorporating a trust model to prevent untrusted nodes from participating in the consensus process, taking into account both the benefits of blockchain technology and the limitations of IoT devices^19^. Therefore, as seen in Fig. 5, a VRF approach is randomly created.

In this configuration, consensus processes ought to be dependent on a reliable node and should not be susceptible to malevolent acts. Nonetheless, the first degree of trust analysis is accomplished by figuring out the global trust value for every blockchain network node while taking local trust into account. Subsequently, the initial node list is divided into a list of consensus nodes and a list of backup nodes using the secure encryption sorting algorithm VRF. The consensus process is limited to nodes that are included in the consensus node list, and a starting node is chosen at random from this list as well.

Table 3 compares the suggested method with consensus algorithms based on different parameters in earlier research. Most papers on consensus, use these indices, as they offer a comprehensive evaluation framework for comparing various consensus algorithms. Additionally, these indices illuminate key aspects of each algorithm’s performance, shedding light on their suitability for different use cases and network environments. Through the analysis of these indicators, researchers and practitioners can make informed decisions about which consensus algorithm best aligns with their specific requirements, whether it be scalability, decentralization, fault tolerance, or efficiency.

**Table 3:** Comparative analysis of proposed algorithm with other existing algorithms.

Parameters	PoS	PoW	PBFT consensus-based lightweight blockchain^19^	DPoS	Multi-leader (quorum-based^18^)	POA^32^	Proposed algorithm
Nodes participating in the consensus process	Variable	High	Variable	Variable	Variable (depends on quorum size)	N/A	Variable
Node reliability assessment	Based on stake (economic interest)	N/A	Typically, reliable	Based on votes (delegated)	Variable (depends on quorum size)	–	Typically, reliable
Network type	Permissioned	Permissionless	Permissioned	Permissioned	Permissioned	Permissioned	Permissioned
Node election fairness	Depends on stake	N/A	Typically, fair	Depends on votes	Depends on quorum size	N/A	Typically, fair
Communication overhead	Moderate to high	High	High	Moderate to high	Variable (depends on quorum size)	Low to moderate	Low to moderate
Number of sent and received messages	Variable	High	High	Variable	Variable (depends on quorum size)	Low to moderate	Low to moderate

### Discussion

The selection of the appropriate algorithm should be based on the characteristics and requirements of the environment and the desired system. These comparisons can assist in deciding on the suitable algorithm for a specific system. Various algorithms have been proposed to ensure consistency in systems and networks. In Table 3, some consensus algorithms have been compared based on various parameters. For example, the communication overhead in these algorithms indicates the number of messages exchanged between nodes and has a direct impact on the performance and efficiency of the consensus process. Proof of Work (PoW) has a high energy consumption and involves the transmission of a large number of messages in the consensus process, resulting in a high communication overhead that may lead to environmental issues. In Proof of Stake (PoS), lower energy consumption and lower communication overhead are imposed. Practical Byzantine Fault Tolerance (PBFT) also involves a high number of messages exchanged between nodes, creating a significant communication overhead. Delegated Proof of Stake (DPoS) reduces communication overhead by minimizing the number of messages compared to PoW and PoS. Multi-Leader (Quorum-Based) depends on the number of nodes and leaders and may introduce a variable amount of communication overhead. Raft limits the number of its messages to a specified number of nodes, leading to reduced communication overhead and improved efficiency^33,34^.

The number of participating nodes in these algorithms indicates the nodes involved in the consensus process and plays a significant role in system security and efficiency^17^. PoW utilizes a large number of nodes in the consensus process, which enhances security but increases communication costs and energy consumption. In PoS, the number of participating nodes may be lower and is determined based on the number of coins held by the nodes. PBFT uses a specific and limited number of nodes for consensus, which is fewer than PoW. DPoS reduces the number of participating nodes, and nodes are selected by other nodes. Multi-Leader (Quorum-Based) requires flexibility in the number of nodes, and its quantity depends on changes. Raft uses a specific and predefined number of nodes for the consensus process, which leads to simplicity and greater adjustability^19^.

The number of sent and received messages in these algorithms indicates the network traffic and the need for communication between nodes. PoW involves a large number of sent and received messages in its computational process, resulting in high energy consumption and delays in consensus. In PoS, the number of messages is lower as the selection of nodes is based on the number of coins they possess. PBFT also involves a high number of messages exchanged among nodes, requiring active communication between nodes. DPoS has fewer messages compared to PoW and PoS since nodes are chosen by other nodes. Multi-Leader (Quorum-Based) determines the required number of messages based on the number of nodes and leaders. Raft limits its messages to a specified number of nodes, which reduces network traffic and improves efficiency. These algorithms represent different methods for selecting nodes to participate in the consensus process. In PoW, nodes are selected randomly, and trust is based on the results of computations and their computational complexity. In PoS, the selection of nodes is based on the number of coins they hold, and trust is established based on the quantity of coins and the performance history of nodes. In PBFT, nodes must be recognized as valid by other nodes, and trust depends on the existence of valid nodes. In DPoS, nodes are selected by other nodes, and trust is established based on the number of selections and the presence of valid nodes. Multi-Leader (Quorum-Based) creates trust in nodes through leaders and some nodes, and the selection of nodes is in the hands of the valid ones. In Raft, trust in nodes is established by the leader, and the selection of nodes is carried out according to the leader’s recommendation.

## Conclusions

The proposed method presents a cutting-edge architecture for delivering medical and healthcare services, incorporating the use of blockchain technology and consensus-based decision-making. This article approach enables a collaborative environment among specialized physicians, allowing them to contribute their expert opinions on patient diagnoses and treatments. The consensus process ensures that the most agreed-upon diagnosis is prescribed to the patient, enhancing the quality and reliability of medical decisions. Physicians and patients register in the system, providing verified identity and professional information, ensuring a secure and reliable network. Patients can choose their treating physician based on their required specialty, with the option to view physicians’ ratings given by other patients. Once the treating physician is selected, patients share their medical history for examination.

The consensus process involves inviting other qualified physicians to participate based on their expertise, and their decisions are calculated using a Weighted Sum formula. During the voting process, participating physicians cast their votes and opinions on the diagnosis, and if the treating physician’s diagnosis receives the majority of votes, it is accepted. Blockchain technology ensures the immutability of all transactions, preventing any tampering or manipulation of medical information, and guaranteeing data integrity and transparency. The proposed method enhances patient confidence, knowing their medical data is securely stored and handled with utmost privacy.

To summarize the limitations and weaknesses of the approach, the following points can be noted:

- *Cost and adoption* Implementing and maintaining a blockchain-based healthcare system can be costly, requiring investment in infrastructure, technology, and ongoing maintenance. Additionally, convincing healthcare providers and patients to adopt the new system may be challenging, especially if they are accustomed to traditional methods of diagnosis and treatment.
- *Privacy concerns* While blockchain technology ensures the immutability of transactions and enhances data security, it also raises privacy concerns. Storing sensitive medical information on a blockchain may expose patients to privacy risks if the data is not adequately protected or if there are vulnerabilities in the system.
- *Scalability* As the system relies on inviting other qualified physicians to participate in the consensus process, scalability could become an issue as the number of participants increases. Managing a large number of physicians and their opinions could become challenging and may slow down the decision-making process.

A concern on the applicability of the proposed approach may be the resistance from doctors towards adopting blockchain technology for managing medical records which may stem from concerns about complexity, potential delays in decision-making, efficiency, resistance to change, patient trust, regulatory compliance, and integration challenges with existing systems. Addressing these concerns requires providing education, training, and transparent communication to demonstrate the benefits of blockchain, alleviate fears, and engage doctors as stakeholders in the adoption process. It’s essential to highlight how blockchain can streamline processes, enhance data integrity, improve efficiency, comply with regulations, and ultimately benefit patient care while addressing any perceived drawbacks or challenges.

## Data Availability

The datasets used and/or analyzed during the current study are available from the corresponding author upon reasonable request.
